# Morphological keys for the identification of Italian phlebotomine sand flies (Diptera: Psychodidae: Phlebotominae)

**DOI:** 10.1186/s13071-014-0479-5

**Published:** 2014-10-17

**Authors:** Filipe Dantas-Torres, Viviana Domenica Tarallo, Domenico Otranto

**Affiliations:** Dipartimento di Medicina Veterinaria, Università degli Studi di Bari, Strada Provinciale per Casamassima, 70010 Valenzano Bari, Italy; Departamento de Imunologia, Centro de Pesquisas Aggeu Magalhães, Recife, Brazil

**Keywords:** Sand flies, Taxonomy, Vectors, Leishmaniasis

## Abstract

**Background:**

Phlebotomine sand flies are small blood-feeding insects of great medical and veterinary significance. Their identification relies basically on the microscopic examination of key morphological characters. Therefore, identification keys are fundamental to any researcher dealing with these insects. The Italian fauna of phlebotomine sand flies consists of eight species (*Phlebotomus perniciosus*, *Phlebotomus perfiliewi*, *Phlebotomus ariasi*, *Phlebotomus neglectus*, *Phlebotomus papatasi*, *Phlebotomus mascittii*, *Phlebotomus sergenti* and *Sergentomyia minuta*), whose morphological delineation may be troublesome for non-taxonomists.

**Methods:**

A total of 8,757 pictures were taken from the 419 selected phlebotomine sand fly specimens collected on different occasions. Twenty-eight characters for the males and 23 for the females were examined, resulting in a database containing over 10,000 entries. Representative phlebotomine sand fly specimens for each species available were selected and relevant characters were drawn with the aid of a camera lucida.

**Results:**

After detailed morphological study of representative specimens, comprehensive identification keys based on key characters (e.g., pharynx and spermathecae of females and male terminalia) were elaborated.

**Conclusions:**

The identification keys provided herein allow the identification of genera and species of phlebotomine sand flies of Italy and they will facilitate future studies on these medically important insects.

## Background

Phlebotomine sand flies (Diptera: Psychodidae: Phlebotominae) are blood-feeding insects of great medico-veterinary significance. Indeed, they are vectors of numerous pathogens to animals and humans, including protozoa, bacteria and viruses [1,2]. For instance, species of the genus *Phlebotomus* are vectors of phleboviruses (e.g., sand fly fever Naples virus, and sand fly fever Sicilian virus) causing the sand fly fever, which is a transient febrile illness that is mainly prevalent in the Mediterranean region [2,3]. Most importantly, phlebotomine sand flies are the biological vectors of *Leishmania* parasites which still cause disfiguring lesions and claim the lives of thousands of dogs and humans each year in more than 90 endemic countries [3].

Both cutaneous and visceral forms of leishmaniasis are quite prevalent in southern Europe [4]. Among other factors, the high prevalence of human and animal leishmaniasis in southern Europe is a consequence of the wide distribution and density of phlebotomine sand fly vectors. Indeed, they are spread throughout southern Europe, particularly in countries such as Portugal, Spain, France, Italy and Greece [4]. For instance, the Italian fauna of phlebotomine sand flies includes eight species, namely, *Phlebotomus perniciosus* Newstead, 1911, *Phlebotomus perfiliewi* Parrot, 1930, *Phlebotomus ariasi* Tonnoir, 1921, *Phlebotomus neglectus* Tonnoir, 1921, *Phlebotomus papatasi* (Scopoli, 1786), *Phlebotomus mascittii* Grassi, 1908, *Phlebotomus sergenti* Parrot, 1917 and *Sergentomyia minuta* (Rondani, 1843) [5-8]. Incidentally, the identification of phlebotomine sand flies in Italy have been based on morphological features of the pharynx of females [9], on the number of Newstead’s spines (=hyaline sensilla) on the third palpal segment [10] and, most frequently, on the morphology of the spermathecae (*receptacula seminis*) of the female [11]. Nonetheless, comprehensive and illustrated identification keys for Italian sand flies are not available in the international literature. Indeed, recent Italian studies have adopted different taxonomic sources. For instance, Rossi et al. [12] and Morosetti et al. [13] have based their identification on French [14] and German [15] works, whereas Tarallo et al. [6] have adopted a paper on the identification of female sand flies of the subgenus *Larroussius* [16] and an Italian book with illustrations for the identification of acari and insects of medical and veterinary significance [17].

Recent studies have demonstrated the usefulness of different genetic markers (e.g., ITS2 and cytb-nd1 regions) for the molecular identification of phlebotomine sand flies [18-20]. In the same way, protein profiling using the matrix-assisted laser desorption/ionization time of flight mass spectrometry (MALDI-TOF MS) has also be proposed as a promising tool for the identification of phlebotomine sand flies [21]. Nonetheless, the identification of these insects is still primarily achieved through the microscopic examination of key morphological characters, including pharynx, spermathecae and cibarium of females as well as male terminalia [22,23]. Thus, morphological keys for the identification of phlebotomine sand flies are pivotal for studies dealing with these insects. In this context, we propose herein identification keys for genera and species of Phlebotominae of Italy.

## Methods

Phlebotomine sand fly specimens used herein were collected at different occasions, in studies conducted in Apulia, Sicily and Basilicata regions, southern Italy [6-8,18]. As a rule, collection sites were selected based on their characteristics, including presence of animals, type of vegetation, and degree of urbanization. Phlebotomine sand flies were collected using ordinary collection methods, such as sticky traps (white paper sheets coated with Castor oil), light traps (model IMT, Byblos per l’Igiene Ambientale di Wehbe Nasser, Cantù, CO, Italy) or mouth aspirators. Phlebotomine sand flies collected with light traps and mouth aspirators were directly preserved in 70% ethanol. Those caught with sticky traps, however, were firstly washed with 90% ethanol, in order to remove excess of oil [5] and then kept in labelled vials containing 70% ethanol.

Before proceeding with species identification, phlebotomine sand flies were examined using a stereomicroscope (Leica Microsystems, MS5, Germany), separated from other insects and according to sex. For mounting on slides, specimens were cleared with 10% potassium hydroxide solution at room temperature for 2 h. The material was then washed with water for 1–2 min, immersed in 10% aqueous solution of glacial acetic acid for 30 min, washed again with water for 30 min and, finally, slide-mounted in Hoyer’s solution as described by Lewis [24]. Species identification was made according to different morphological keys, species descriptions and other identification resources [14,16,17,25].

Out of about 16,500 phlebotomine sand flies examined over the past 10 years, representative specimens of each species were selected and further studied morphologically. Specimens of both sexes (i.e., 233 males and 186 females) were selected based on conservation status and quality of the clarification. In some cases, all insects of a given species (e.g., *P. sergenti*) or of a specific sex (e.g., *P. neglectus* female) were used, due to the limited number of specimens available. Several morphological characters were examined, but only key characters (e.g., pharynx and spermathecae of females and terminalia of males) were considered during the preparation of the identification keys. Incidentally, these characters were those reported in the keys proposed by Lewis [24].

Representative phlebotomine sand fly specimens for each species available were selected and relevant characters were drawn with the aid of a camera lucida (Leica Microsystems, L 3/20, Germany). The pencil drawings were scanned, the resulting files were imported into Adobe Illustrator C6 and the line drawings were made using a digitiser board (WACOM Intuous 5 touch PTH-650, Wacom Europe GmbH, Germany). Voucher phlebotomine sand fly specimens are deposited in the Laboratory of Parasitology and Parasitic Diseases at the Department of Veterinary Medicine, University of Bari, Italy.

## Results and discussion

**Figure 1:**
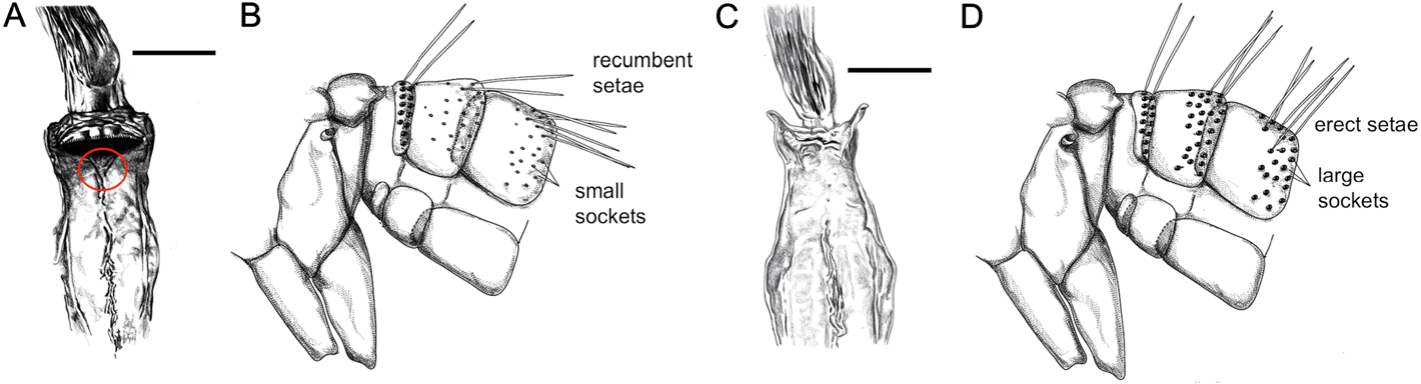
**Abdominal tergites and cibarium.** *Sergentomyia* sp.: cibarium **(A)** (scale bar =50 μm) and abdominal tergites 1–3 **(B)** (not to scale). *Phlebotomus* sp.: cibarium **(C)** (scale bar =50 μm) and abdominal tergites 1–3 **(D)** (not to scale).

**Figure 2:**
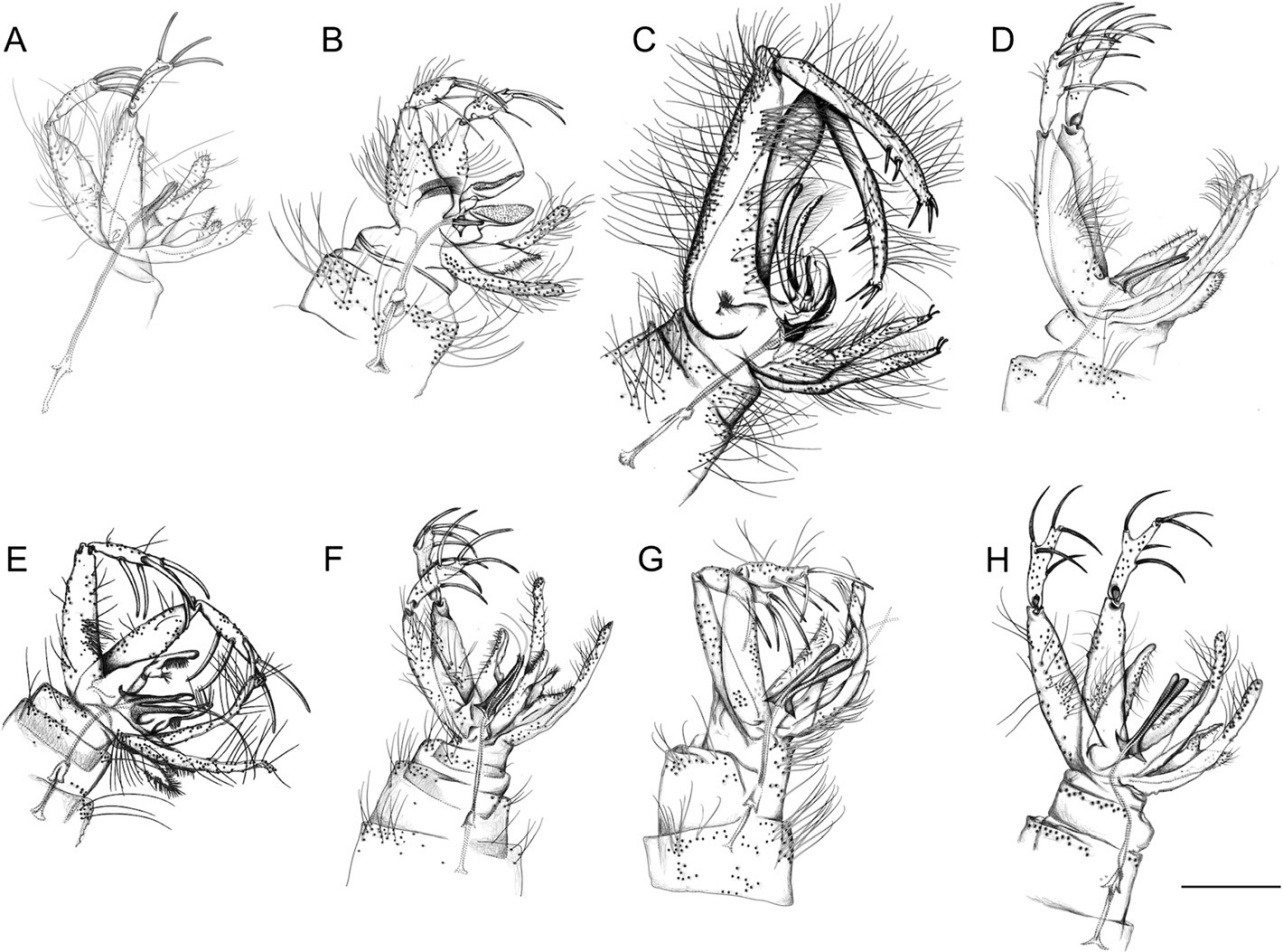
**Terminalia of male phlebotomine sand flies. A**, *Sergentomyia minuta*. **B**, *Phlebotomus sergenti*. **C**, *Phlebotomus papatasi*. **D**, *Phlebotomus mascittii*. **E**, *Phlebotomus perfiliewi*. **F**, *Phlebotomus perniciosus*. **G**, *Phlebotomus ariasi*. **H**, *Phlebotomus neglectus*. Scale bar =200 μm.

**Figure 3:**
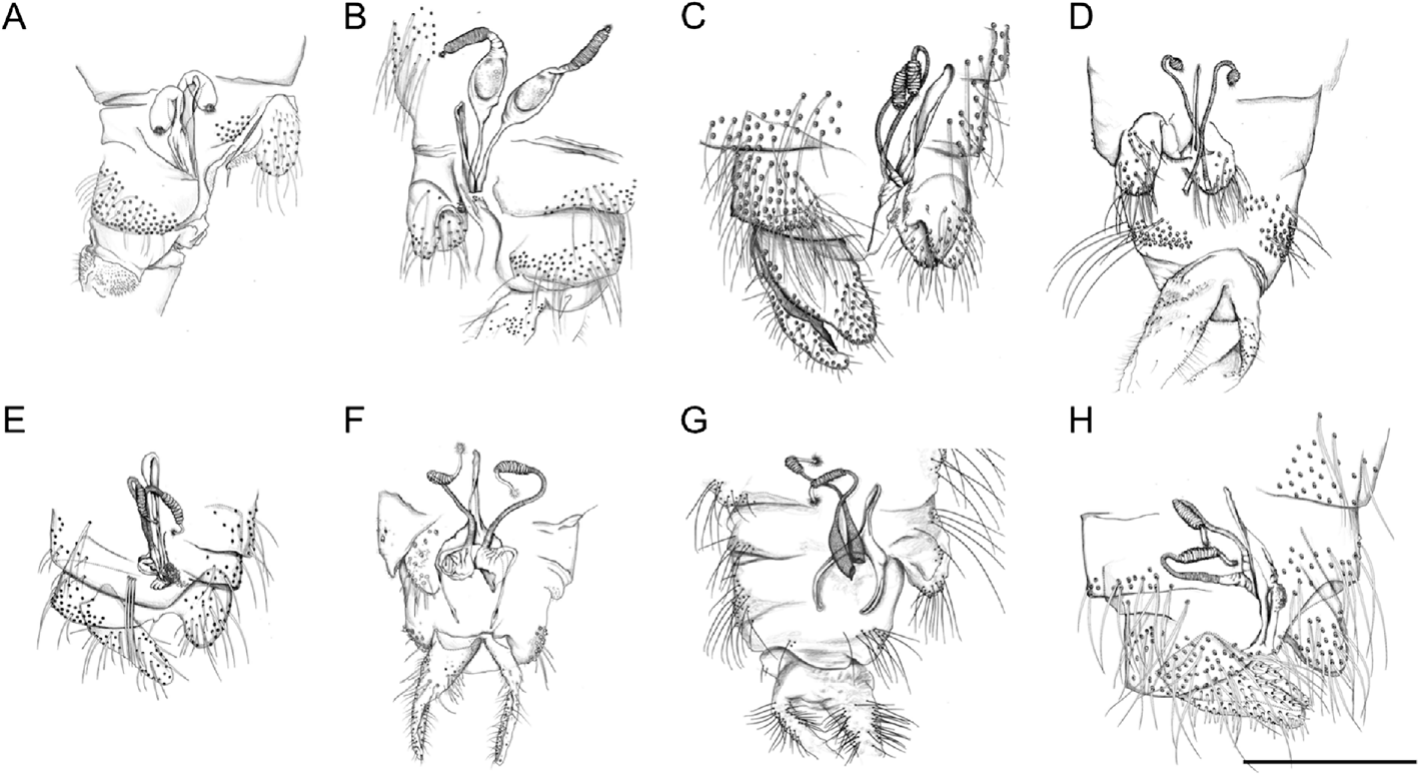
**Spermathecae of female phlebotomine sand flies. A**, *Sergentomyia minuta*. **B**, *Phlebotomus mascittii*. **C**, *Phlebotomus papatasi*. **D**, *Phlebotomus sergenti*. **E**, *Phlebotomus perniciosus*. **F**, *Phlebotomus perfiliewi*. **G**, *Phlebotomus ariasi*. **H**, *Phlebotomus neglectus*. Scale bar =200 μm.

**Figure 4:**
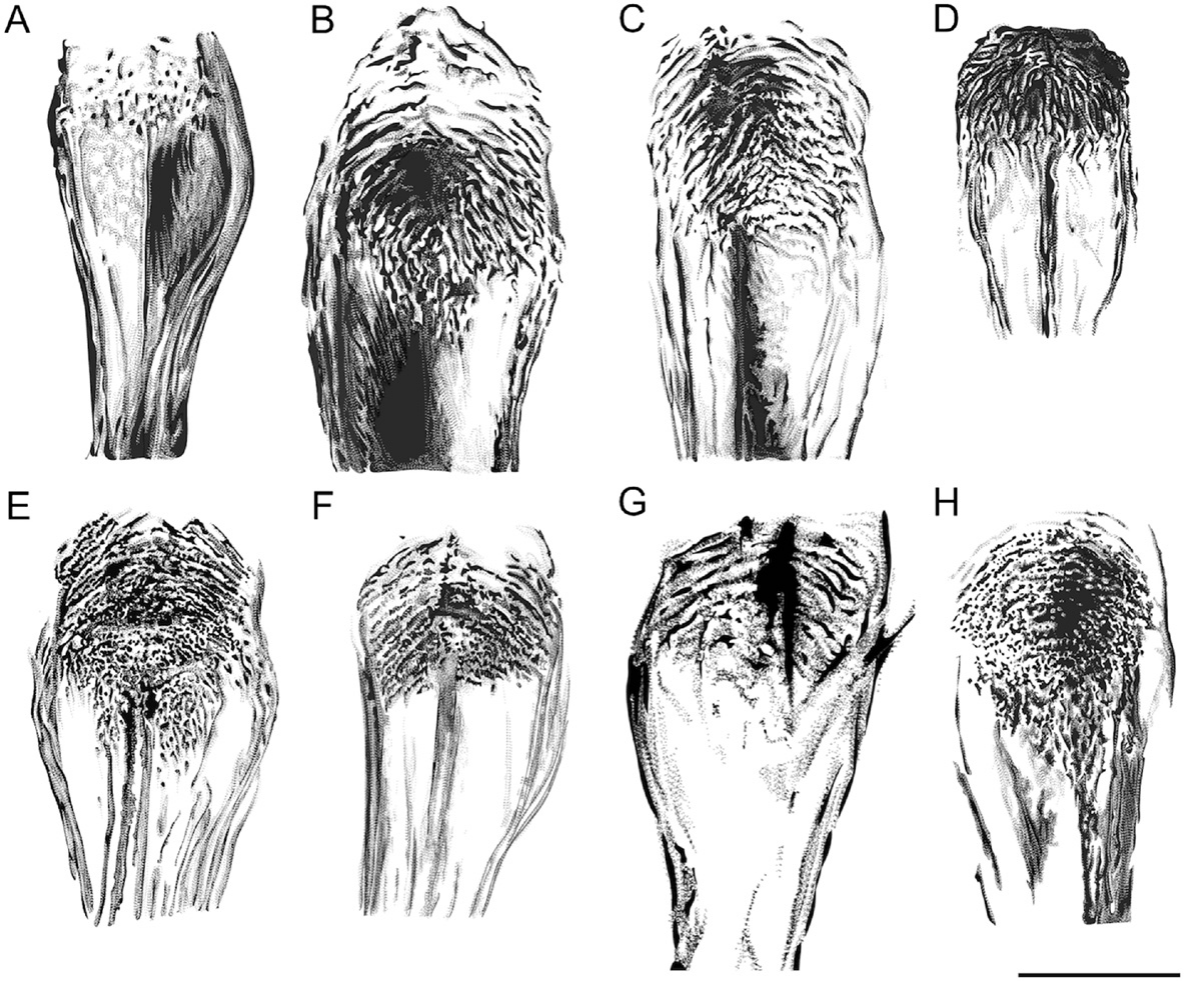
**Pharynx of female phlebotomine sand flies. A**, *Sergentomyia minuta*. **B**, *Phlebotomus mascittii*. **C**, *Phlebotomus papatasi*. **D**, *Phlebotomus sergenti*. **E**, *Phlebotomus perniciosus*. **F**, *Phlebotomus perfiliewi*. **G**, *Phlebotomus ariasi*. **H**, *Phlebotomus neglectus*. Scale bar =50 μm.

After detailed morphological study of representative specimens, comprehensive identification keys for genera and species of phlebotomine sand flies of Italy were elaborated (Tables 1, 2 and 3; Figures 1, 2, 3 and 4).

**Table 1:** **Key to the genera of Phlebotominae of Italy**

1.	Cibarial teeth in a transverse row and pigment patch usually present (Figure 1A; red circle). Hind end of abdominal tergites 2–6 with all or most setae recumbent, which arise from small sockets as compared with those on tergite 1 (Figure 1B). Male style with 3 apical and 1 sub-apical long spines developed and 1 more basal setiform spine (accessory seta) (Figure 2A). Female spermatechae smooth (Figure 3A)	*Sergentomyia* ^*a*^
	Cibarial teeth and pigment patch usually absent (Figure 1C). Hind end of abdominal tergites 2–6 with many erect setae, which arise from large sockets of the same size as those on tergite 1 (Figure 1D). Male style with 2 long apical spines (Figure 2B,D-H) or 3 short apical spines (Figure 2C) and setiform spine absent. Female spermatechae with superficial striation (Figure 3B) or annulated (Figure 3C-H)	*Phlebotomus*

^a^ *Sergentomyia minuta* (Figures 1A-B, 2A, 3A, 4A) is the only species of this genus reported in Italy so far.

**Table 2:** **Key to the males of** ***Phlebotomus*** **of Italy**

1.	Coxite (=basistyle) with bristles in the basal region	2
	Coxite without bristles in the basal region	3
2.	Style (=dististyle) short with 4 long spines: 2 apical and the internal more basal than the external spine. Paramere without dorsal ramification. Surstyle (=lateral lobe) without short distal spines (subgenus *Paraphlebotomus*)	*P. sergenti* (Figure 2B)
	Style with 5 short spines: 3 apical and 2 external spines in its apical third. Paramere with 2 dorsal ramifications. Surstyle with 2 short distal spines (subgenus *Phlebotomus*)	*P. papatasi* (Figure 2C)
3.	Aedeagus with the distal region tapered and cup-like expansion at the anterior end of genital pump thin-walled and colourless (subgenus *Transphlebotomus*)	*P. mascittii* (Figure 2D)
	Aedeagus with the distal region bifid or rounded (subgenus *Larroussius*)	4
4.	Aedeagus with colourless plate (transparent process) on terminal portion	*P. perfiliewi* (Figure 2E)
	Aedeagus without colourless plate on terminal portion	5
5.	Aedeagus bifid at the end	*P. perniciosus* (Figure 2F)
	Aedeagus not bifid at the end	6
6.	Adeagus clapper-like, with moderate subapical expansion	*P. ariasi* (Figure 2G)
	Adeagus with drumstick-like tip	*P. neglectus* (Figure 2H)

**Table 3:** **Key to the females of** ***Phlebotomus*** **of Italy**

1.	Spermathecae not ringed, with transverse striations often in distal part. Pharynx with large irregular teeth (subgenus *Transphlebotomus*)	*P. mascitti* (Figs. 3B and 4B)
	Spermathecae ringed. Pharynx not as above	2
2.	Spermathecae without neck	3
	Spermathecae with long finger-like neck (subgenus *Larroussius*)	4
3.	Spermathecae with 8–12 rings (apical segment short). Pharyngeal armature not extending beyond its posterior third, with scaly teeth arranged into a wide-meshed network (subgenus *Phlebotomus*)	*P. papatasi* (Figs. 3C and 4C)
	Spermathecae with 4–5 rings. Pharyngeal armature occupying about a quarter length of pharynx, with few large teeth directed backward (subgenus (subgenus *Paraphlebotomus*)	*P. sergenti* (Figs. 3D and 4D)
4.	Spermathecae with lateral structures at the base of the ducts	5
	Spermathecae without lateral structures at the base of the ducts	6
5.	Spermathecae with 8–12 rings, with thin neck and small, rounded head. Pharyngeal armature occupying more than a quarter length of pharynx, with teeth arranged disorderly	*P. perniciosus* (Figs. 3E and 4E)
	Spermathecae with 12-16 rings, small neck that narrows before the small, oval head. Pharyngeal armature about a quarter (or less) length of pharynx with teeth ending anteriorly in a clear line of demarcation	*P. perfiliewi* (Figs. 3F and 4F)
6.	Spermathecae with 8–16 rings, with spermathecal ducts sac-like. Pharyngeal armature occupying less then a third of pharynx	*P. ariasi* (Figs. 3G and 4G)
	Spermathecae with 12–16 rings, with neck surrounded by a sleeve. Pharyngeal armature occupying more than a third of pharynx	*P. neglectus* (Figs. 3H and 4H)

Identification keys are fundamental for anyone dealing with insects of medical and veterinary significance, such as phlebotomine sand flies. They are intended to provide a guide for those interested to identify field-collected specimens obtained for distinct purposes and different kind of studies (e.g., seasonality, vectorial role and taxonomy). Indeed, identification keys, especially those accompanied by line drawings illustrating taxonomically relevant characters, are useful for species identification of phlebotomine sand flies [26].

Filippo Bonanni, an Italian Jesuit scholar, published in 1691 the first illustration of a phlebotomine sand fly. Later on, in 1786, the Italian naturalist Giovanni Antonio Scopoli described the species *Bibio papatasi* (later replaced in the genus *Phlebotomus*), the first phlebotomine sand fly ever described [25]. In the same way, at the end of the 19th and beginning of the 20th centuries, respectively, Rondani and Grassi described new species based on material collected in Italy [25]. Interestingly, in spite of the long tradition of Italy in the field of phlebotomine sand fly and leishmaniasis research, the identification of these insects in Italian studies has mostly been based on old keys and/or on an Italian book for the identification of acari and insects of medical and veterinary significance [17]. To the authors’ knowledge, before the present work, no keys for the identification of genera and species of phlebotomine sand flies of Italy were available in the international literature.

## Conclusions

In conclusion, the present paper provides identification keys for genera and species of phlebotomine sand flies found in Italy, which will facilitate future studies on these medically important insects. These simplified keys, along with the line drawings provided herein are intended for anyone dealing with sand fly identification in Italy and may also be useful for those working in other Mediterranean countries, as most of the species found in Italy are also prevalent in countries such as Spain, Portugal and Greece [4].

## References

[1] Dantas-Torres F, Solano-Gallego L, Baneth G, Ribeiro VM, de Paiva-Cavalcanti M, Otranto D (2012). Canine leishmaniosis in the Old and New Worlds: unveiled similarities and differences. Trends Parasitol.

[2] Maroli M, Feliciangeli MD, Bichaud L, Charrel RN, Gradoni L (2013). Phlebotomine sandflies and the spreading of leishmaniases and other diseases of public health concern. Med Vet Entomol.

[3] Alvar J, Vélez ID, Bern C, Herrero M, Desjeux P, Cano J, Jannin J, den Boer M, WHO Leishmaniasis Control Team (2012). Leishmaniasis worldwide and global estimates of its incidence. PLoS ONE.

[4] Ready PD (2010). Leishmaniasis emergence in Europe. Euro Surveill.

[5] Maroli M, Fausto AM (1986). Metodi di Campionamento e Montaggio dei Phlebotomi (Diptera: Psychodidae).

[6] Tarallo VD, Dantas-Torres F, Lia RP, Otranto D (2011). Phlebotomine sand fly population dynamics in a leishmaniasis endemic peri-urban area in southern Italy. Acta Trop.

[7] Dantas-Torres F, Tarallo VD, Falchi A, Lia RP, Otranto D (2014). **Ecology of phlebotomine sand flies****and*****Leishmania infantum*****infection in a rural area of southern Italy.**. Acta Trop.

[8] Gaglio G, Brianti E, Napoli E, Falsone L, Dantas-Torres F, Tarallo VD, Otranto D, Giannetto S (2014). Effect of night time-intervals, height of traps and lunar phases on sand fly collection in a highly endemic area for canine leishmaniasis. Acta Trop.

[9] Corradetti A, Neri I, Verolini F, Palmieri G, Proietti AM (1961). Technical procedure for the study of the pharynx of phlebotomine sandflies and description of the pharynx of Italian sandflies. Parassitologia.

[10] Biocca E, Coluzzi A, Costantini R (1977). Osservazioni sulla attuale distribuzione dei flebotomi italiani e su alcuni caratteri morfologici differenziali tra le specie del sottogenere *Phlebotomus* (*Larroussius*). Parassitologia.

[11] Maroli M, Bigliocchi F, Khoury C (1994). I flebotomi in Italia: osservazioni sulla distribuzione e sui metodi di campionamento. Parassitologia.

[12] Rossi E, Rinaldi L, Musella V, Veneziano V, Carbone S, Gradoni L, Cringoli G, Maroli M (2007). Mapping the main *Leishmania* phlebotomine vector in the endemic focus of the Mt. Vesuvius in southern Italy. Geospat Health.

[13] Morosetti G, Bongiorno G, Beran B, Scalone A, Moser J, Gramiccia M, Gradoni L, Maroli M (2009). Risk assessment for canine leishmaniasis spreading in the north of Italy. Geospat Health.

[14] Léger N, Pesson B, Madulo-Leblond G, Abonnenc E (1983). Sur la différenciation des femelles du sous-genre *Laroussius* Nitzulescu, 1931 (Diptera-Phlebotomidae) de la region méditerranéenne. Ann Parasitol Hum Comp.

[15] Theodor O (1958). Psychodidae-Phlebotominae. Die Fliegen der Palearktischen Region, 9c, Schweiterbart’sche Verlagsbuchhandlung, Stuttgart (D).

[16] Killick-Kendrick R, Tang Y, Killick-Kendrick M, Sang DK, Sirdar MK, Ke L, Ashford RW, Schorscher J, Johnson RH (1991). The identification of female sandflies of the subgenus *Larroussius* by the morphology of the spermathecal ducts. Parassitologia.

[17] Romi R, Khoury C, Bigliocchi F, Maroli M (1994). Schede Guida su Acari e Insetti di Interesse Sanitario.

[18] Dantas-Torres F, Latrofa MS, Otranto D (2010). Occurrence and genetic variability of *Phlebotomus papatasi* in an urban area of southern Italy. Parasit Vectors.

[19] Latrofa MS, Dantas-Torres F, Weigl S, Tarallo VD, Parisi A, Traversa D, Otranto D (2011). Multilocus molecular and phylogenetic analysis of phlebotomine sand flies (Diptera: Psychodidae) from southern Italy. Acta Trop.

[20] Latrofa MS, Annoscia G, Dantas-Torres F, Traversa D, Otranto D (2012). Towards a rapid molecular identification of the common phlebotomine sand flies in the Mediterranean region. Vet Parasitol.

[21] Dvorak V, Halada P, Hlavackova K, Dokianakis E, Antoniou M, Volf P (2014). Identification of phlebotomine sand flies (Diptera: Psychodidae) by matrix-assisted laser desorption/ionization time of flight mass spectrometry. Parasit Vectors.

[22] Abonnenc E (1972). Les phlebotomes de la region Ethiopienne (Diptera, Psichodidae). Mem Off Rech Sci Tech Outre-Mer.

[23] Rahola N, Depaquit J, Makanga BK, Paupy C (2013). *Phlebotomus* (*Legeromyia*) *multihamatus* subg. nov., sp. nov. from Gabon (Diptera: Psychodidae). Mem Inst Oswaldo Cruz.

[24] Lewis DJ, Smith KGV (1973). Phlebotomidae and Psychodidae (Sand-Flies and Moth-Flies). Insect and Other Arthropods of Medical Importance.

[25] Lewis DJ (1982). A taxonomic review of the genus *Phlebotomus* (Diptera: Psychodidae). Bull Br Mus Nat Hist.

[26] Shimabukuro PHF, Tolezano JE, Galati EAB (2011). Chave de identificação ilustrada dos Phlebotominae (Diptera, Psychodidae) do estado de São Paulo, Brasil. Pap Avulsos Zool.

